# Respiratory Failure Secondary to Human Metapneumovirus Requiring Extracorporeal Membrane Oxygenation in a 32-Month-Old Child

**DOI:** 10.1155/2012/268074

**Published:** 2012-05-22

**Authors:** Abha Gupta, Melania Bembea, Anna Brown, Courtney Robertson, Lewis Romer, Ronald D. Cohn

**Affiliations:** ^1^Department of Pediatrics, Johns Hopkins Hospital, Baltimore, MD 21287, USA; ^2^Department of Anesthesiology and Critical Care Medicine, Johns Hopkins Hospital, Baltimore, MD 21287, USA; ^3^McKusick-Nathans Institute of Genetic Medicine, Johns Hopkins University School of Medicine, Broadway Research Building, 733 Nth Broadway, Room 529, Baltimore, MD 21205, USA

## Abstract

Human metapneumovirus (HMPV) is a common virus that can cause respiratory problems ranging from mild upper respiratory tract disease to respiratory failure requiring mechanical support. Here, we report a case of a 32-month-old male with a previous history of asthma, who developed respiratory failure two weeks after onset of cough and rhinorrhea and required extracorporeal membrane oxygenation (ECMO) for 9 days after failing high-frequency oscillatory ventilation (HFOV). To our knowledge, this is the oldest reported pediatric patient with respiratory failure secondary to human metapneumovirus that did not respond to mechanical ventilation. This case highlights three critical points: the potentially fatal causative role of HMPV in respiratory failure in an older pediatric age group of immunocompetent hosts, the importance of early recognition of impending respiratory failure, and the timely utilization of ECMO.

## 1. Background

Human metapneumovirus, an RNA virus in the paramyxovirus family, was first isolated from 28 patients in The Netherlands in 2001. The spectrum of disease ranges from mild upper respiratory tract disease to severe bronchiolitis and pneumonia requiring mechanical ventilation [1]. 

There have been four reported cases of pediatric patients with HMPV-induced respiratory failure who did not respond to mechanical ventilation: a 17-month-old with acute lymphocytic leukemia receiving chemotherapy who died of respiratory failure [2]; a three-month-old infant with history of premature birth at 27 weeks who survived after ECMO [3]; a nine-month-old infant on immunosuppressive therapy after a liver transplant for biliary atresia who also survived after ECMO [4], and a two-year-old previously healthy immunocompetent girl who died of pulmonary hemorrhage while on conventional ventilation [5]. Here, we present the case of a thirty-two-month-old immunocompetent boy with HMPV-induced respiratory failure and a history of asthma who was supported with ECMO and survived.

## 2. Case Presentation

The patient is a 32-month-old male with a history of asthma who had been born at 36 weeks gestation via an unremarkable induced vaginal delivery secondary to oligohydramnios, with a birth weight of 5 pounds 12 ounces. The patient's asthma history is significant for multiple emergency room visits and one prior non-PICU admission for an asthma exacerbation. His maintenance medical regimen includes daily inhaled corticosteroids and albuterol as needed. The patient lives with his mother, father, an older sister who is healthy, a maternal grandmother who smokes in the house, and a cat. 

**Figure 1:**
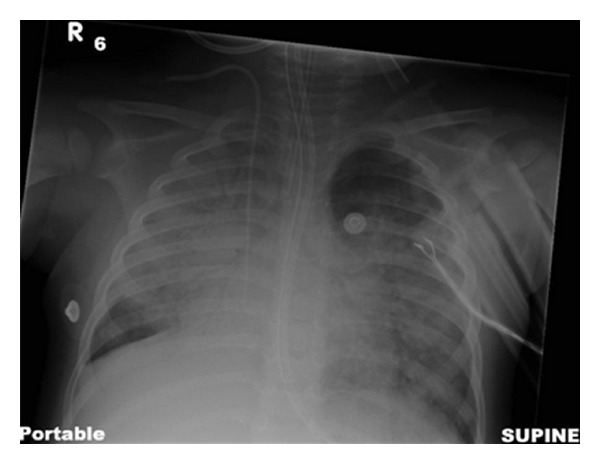
Initial chest radiograph prior to ECMO initiation.

The patient presented to a nearby emergency room seven days prior to presentation with worsening respiratory distress in the context of two weeks of upper respiratory symptoms and diarrhea. On initial presentation at the nearby emergency room, he was diagnosed with bilateral pneumonia and treated as an outpatient with amoxicillin, azithromycin, oral steroids, and nebulized albuterol treatments. Four days after the initial emergency room visit, his respiratory symptoms progressed, necessitating emergency intubation and high frequency oscillatory ventilation at the outside hospital. The patient was positive for human metapneumovirus and had healthy appearing airways on bronchoscopy. After three days of progressive worsening on increased respiratory support, the patient was transferred to the JHH Pediatric Intensive Care Unit from an outside hospital and venovenous extracorporeal membrane oxygenation was initiated. Please refer to Figure 1 for the chest radiograph prior to ECMO initiation. 

The patient was initially treated with vancomycin, piperacillin/tazobactam, and fluconazole for presumed superinfection in addition to the HMPV pneumonia. After the blood cultures were negative for bacteria, fungi, and viruses for 48 hours, vancomycin and fluconazole were discontinued. However, due to the severity of his illness and concern for bacterial suprainfection, he completed an 11-day course of piperacillin/tazobactam for presumptive pneumonia. Due to decreasing urine output and concern for fluid overload compromising his respiratory status, the patient was started on continuous venovenous hemofiltration with a positive response, and he was subsequently transitioned to a furosemide infusion. He also required a nicardipine infusion for elevated blood pressures. Heparin anticoagulation and routine blood product transfusions were used during the ECMO run as per protocol. 

The patient progressed favorably from a respiratory perspective and was successfully decannulated on day 9 of ECMO, extubated 4 days following ECMO, and transitioned to room air 9 days following extubation. 

Given the unusual clinical course, an immunodeficiency workup to further investigate possible reasons for the development of respiratory failure in an otherwise healthy host was performed. T cell subsets were within normal limits. The remainder of his hospital course on the floor was notable for a successful diuretic wean, normalization of his work of breathing, cleared lung exam without coarseness, subglottic edema with intact vocal cords noted on bedside flexible laryngoscopy, and significant strides in physical and speech therapy. 

## 3. Conclusion

Since its isolation in 2001 [1], HMPV has been implicated as a significant cause of hospitalization in infants and young children. The literature documents detection of HMPV in 1.5–43.0% of children with acute respiratory infections [6]. The most commonly noted clinical manifestations include cough, coryza, fever, irritability, anorexia, wheezing, diarrhea, and vomiting, [7] with a wide spectrum of disease ranging from mild upper respiratory tract disease to severe respiratory distress requiring mechanical ventilation [1].

Because young children have smaller airways, decreased respiratory reserve, increased oxygen consumption and metabolic demands, and less robust compensatory mechanisms, it is important to be cognizant that this population can progress to severe respiratory failure more quickly than their older counterparts. In the case of our patient, he required emergency intubation after failing four days of outpatient therapy for bilateral pneumonia. It is possible that if he had been brought to medical attention during the four days after being seen in the emergency department those signs of early respiratory distress secondary to HMPV, such as increasing tachypnea, retractions, nasal flaring, and tachycardia, would have been recognized. Earlier diagnosis may have enabled a favorable response to less invasive therapies and in turn prevented the need for the escalation of respiratory support with ECMO.

Given the severity of his respiratory failure at presentation, the patient was promptly placed on mechanical ventilation and soon thereafter escalated to ECMO. Evidence suggests that early ECMO intervention in children with reversible respiratory failure may prevent ventilator-associated lung injury and may improve survival [8, 9]. Although further research is needed to identify which patients are most likely to benefit and determine the optimal timing for initiating ECMO, our case indicates that ECMO was a successful rescue strategy in this case. 

In summary, the goals of this case report are as follows: (1) alert clinicians to the possibility of HMPV as a cause of respiratory failure in an older age group of immunocompetent pediatric hosts than previously considered, particularly those with underlying airway disease such as asthma, (2) highlight the importance of early recognition of respiratory failure and the role that timely appreciation of this problem may play in modulating the need for escalation in respiratory support, (3) suggest that timely utilization of ECMO was a key player in the survival of our patient and that it should be considered in similar patients with reversible respiratory failure.
